# Supplementary material for: Are carbon emissions trading and green financial instruments synergistic? -Comprehensive quantitative research based on content analysis
Source: PLoS One. 2024 Mar 7;19(3):e0298601. doi: 10.1371/journal.pone.0298601 (PMC10919601; doi:10.1371/journal.pone.0298601)
Supplement: S1 Data — (ZIP) [file pone.0298601.s001.zip › Data/地级市Z_F工作报告.zip!/║╙─╧/┐¬╖Γ╩╨/└┤╫╘╣1⁄2╓┌║┼í╛┬φ┐╦╩2╛▌═°í┐_2007.docx]

各位代表：

　　现在，我代表市人民政府，向大会作政府工作报告，请予审议，并请各位政协委员提出意见。

　　一、2006年工作回顾

　　2006年，市政府在省委、省政府和市委的正确领导下，在市人大、市政协的监督、帮助和支持下，以科学发展观为指导，努力构建和谐社会，抢抓省委、省政府加快中原崛起、推进郑汴一体化发展的重大机遇，团结带领全市人民开拓进取，扎实工作，较好地完成了市十二届人大三次会议确定的各项任务，实现了“十一五”规划的良好开局。

　　——国民经济持续增长。全市生产总值完成472.4亿元，比上年增长13.1%，其中第一、第二、第三产业增加值分别达到131.2亿元、197.9亿元、143.3亿元，分别增长8.9%、15.7%、13.9%，人均生产总值近1万元；财政总收入完成53.9亿元，增长26%，总支出53.7亿元，增长25.9%；一般预算收入完成16.3亿元，增长16.3%，其中市本级完成7.2亿元，增长18.5%；全社会固定资产投资完成166.3亿元，增长38.5%；社会消费品零售总额177.5亿元，增长15.1%；城镇居民家庭人均可支配收入8286元，增长14.8%；农民人均纯收入3172元，增长16.9%；金融机构各项存款余额359亿元，增长13.9%；贷款余额229.5亿元，增长12.6%。

　　——工业强市成效进一步显现。工业布局进一步优化。规划并开始建设了杏花营高科技工业区、汪屯精细化工区、边村重工业区三个工业区，五县工业区建设初具规模。纺织、食品、化工、医药、机械设备制造等产业优势更加明显，五大产业增加值占规模以上工业增加值的比重达到57%。空分设备、空间结构、预应力、汽车配件等产业集群逐步形成，产业竞争能力进一步提高。工业发展后劲不断增强。新开工和续建重点工业项目53个，累计完成投资26.9亿元，其中投资3000万元以上的重点工业项目竣工25个，完成投资17亿元。工业运行速度和效益进一步提高。全市工业增加值完成179.7亿元，增长16.6%，其中规模以上工业增加值完成100.1亿元，增长22%，实现利税39.7亿元，增长77%。

　　——社会主义新农村建设扎实起步。按照“规划先行、突出重点、搞好试点、扎实推进”的总体思路，围绕发展农村经济和促进农民增收，开展了新农村建设31个示范村和300个整治村试点工作。全市粮、棉、油总产量分别达到233.8万吨、7万吨和34.4万吨，增长15.1%、16.5%和25.2%；肉、蛋、奶产量分别达到40.9万吨、24.5万吨和13.6万吨，增长8.6%、8.3%和50.9%。财政支农力度进一步加大。各级财政用于改善农村生产生活条件的支出达14.5亿元。对种粮农民实行粮食直补、良种补贴、农机具购置补贴、农资综合直补等各项措施全部落实到位，共补贴资金1.7亿元。加大扶贫开发力度，完成了2.6万人脱贫。认真执行“两免一补”政策，发放资金5448万元，资助农村学生48万人次。农业结构调整深入推进。新发展优质专用小麦194万亩、优质专用玉米103万亩、优质水稻12万亩，农产品优质率达到39%；累计建成无公害农产品生产基地29个，总面积49万亩；农业产业化龙头企业达到31个，农村专业合作组织发展到900多个；畜牧业规模养殖比重进一步提高，新发展养殖小区75个，重大动物疫病得到有效控制。农村生产生活条件不断改善。投资3.2亿元，完成了821个村1017公里“村村通”工程，建成了80个乡镇客运站，解决了13万人的饮水安全问题，改建24所乡镇卫生院，新建沼气池3.1万座。实施“万村千乡市场工程”，新改建连锁农家店769个，建成邮政服务网点1800个。农村通信、文化等社会事业取得了新的进展。

　　——各项改革不断深化。国有企业改革继续推进。完成了铁塔橡胶集团、宏达纺机公司、农药厂、涤纶厂等企业国有产权的整体转让，盘活资产4.2亿元，4500多名职工得到妥善安置；无线电一厂、针织内衣厂等企业的破产清算和职工安置工作进展顺利。农村综合改革深入推进。巩固乡镇机构改革成果，清理涉及乡镇的一票否决和评比达标项目，切实减轻乡镇负担；农村义务教育体制进一步完善，提高了农村中小学公用经费标准，建立了农村中小学校舍维修改造长效机制；以清收盘活不良贷款为重点，农村信用社改革不断深化。分配制度改革有序展开。稳步实施公务员法，认真做好公务员登记、工资制度改革和规范津贴补贴工作；近11万名企业离退休人员养老金调整工作如期完成，人均月增资191元，超过全省平均水平25元。其他领域改革取得新成效。财政国库管理制度改革步伐加快，市级已全面推行，县级改革范围进一步扩大；行政审批制度改革继续深化，行政审批事项精简至199项，为全省省辖市最少；干线公路养护体制改革顺利完成；交通规费征稽体制基本理顺；事业单位改革、旅游景区管理体制改革和市政公用行业改革进展顺利。

　　——对外开放力度进一步加大。招商引资成效显著。全年引进省外项目301个，合同资金总额165.9亿元，到位资金68亿元，增长52.9%；新批准外商投资企业16家，合同利用外资1.53亿美元，增长146%，实际利用外资3148万美元，增长37%。国内500强企业已有中国电力投资集团、安阳钢铁集团、平煤集团、深圳能源集团、晋煤集团、雨润集团和永煤集团等7家企业来我市投资发展。进出口贸易增长加快。全市进出口贸易额完成1.35亿美元，增长25%，其中出口7561万美元，增长11.3%。对外交流与合作进一步扩大。成功举办了中国开封第24届菊花花会；参加了2006杭州世界休闲博览会，开封馆以丰富多彩的展览内容和别具特色的文化活动被评为最佳展馆。与杭州市缔结为友好城市，与澳大利亚温格卡瑞比郡建立了友好合作关系。

　　——重点项目建设进展顺利。全市113个重点建设项目完成投资65.9亿元，其中省定13个重点项目完成投资30亿元。开封炭素年产2.2万吨超高功率石墨电极、申丰公司10万锭纺织等一批重点项目竣工投产，郑开大道、大广高速开封段和黄河特大桥建成通车，环城公园一期工程、大广高速东连接线拓宽改造完工，中原皓月现代肉牛产业工程、晋开“1830”工程、汇源三期、各县污水处理厂等项目建设进展顺利。

　　——城镇化进程加快推进。编制完成了汴西新区概念规划和郑开大道两侧功能区规划，启动宋都古城风貌保护与重现工程，加强城市基础设施建设，建成了黄河大街北段道路工程，全市城镇化率达到34.2%，比上年提高1.6个百分点。郑汴一体化发展迈出实质性步伐。与郑州市签署了《关于建立协调发展机制 加快推进郑汴一体化的协定》。郑汴城际公交已经开通，郑开大道两侧产业布局逐渐展开，电信同城、旅游同城、商务同城、金融同城以及面向两市市民的互惠措施正在实施。汴西新区建设全面启动。投资7.2亿元，建成了宋城路西延3.8公里和区内1—6号路南段工程，管网、电网建设已经启动。汴西新区已进驻工业项目32个，总投资64.2亿元；已开工项目18个，总投资25.2亿元。小城镇建设步伐加快。完成了8个省定重点镇和部分乡镇的新一轮规划编制工作。加快县城和小城镇发展，促进企业向城镇集中，人口向城镇聚集，县以下建制镇常驻人口达到78.9万人，增长2.2%。

　　——文化旅游产业发展加快。推进文化体制改革，对市电影公司、人民会场、大众娱乐城等6家单位进行重组，成立了开封市影视娱乐集团；依托开封日报社，组建了神州圣火文化传播有限公司，扩大了开封报业“两报一网”的影响力。认真做好文物保护和景区创优工作，尉氏兴国寺塔、河南留学欧美预备学校旧址、刘青霞故居等6处文物被列为全国重点文物保护单位；开封府、铁塔公园和中国翰园碑林被评为国家4A级景区；朱仙镇木版年画入选首批国家级非物质文化遗产名录。加强旅游宣传和推介，旅游年票发行工作已经启动，全年累计接待游客1891万人次，实现旅游总收入60.5亿元，分别增长15.2%和19.6%。

　　——各项社会事业全面发展。深入实施科教兴汴和人才强市战略，科学技术研究与推广步伐加快，科技对经济增长的支撑作用进一步增强，与中国农大合作建设的现代农业示范工程进展顺利。“两基”成果进一步巩固，新增市二十五中、尉氏三中、杞县高中、兰考一中4所省级示范性高中，职业教育、成人教育和高等教育发展加快。加大公共卫生投入，市疾病预防控制中心建成投用，对重大疾病的预防控制能力增强；扩大农村合作医疗试点，尉氏县、兰考县新型农村合作医疗参合率达到76.2%。全民健身活动深入开展，参加省十运会取得良好成绩。稳定低生育水平，人口自然增长率控制在5‰以下，尉氏县被评为全国计划生育优质服务先进县。精神文明建设取得丰硕成果，窗口行业规范管理优质服务竞赛活动深入开展，科技、文化、卫生“三下乡”和科教、文体、卫生、法律“四进社区”活动扎实有效。水、大气污染治理取得新成效，生态环境进一步改善。新闻出版、广播电视、质量技术监督、食品药品监督、统计、审计、电力、电信、气象、烟草、保险、外事侨务、对台事务、民族宗教、社会科学、地方志、档案管理、地震监测、人民防空等工作都取得了新的成绩。

　　——和谐开封建设取得明显成效。就业再就业工作取得新进展。城镇新增就业7.7万人，下岗失业人员再就业3.9万人，其中“4050”人员1.4万人，市区基本消除了“零就业”家庭。新发放小额担保贷款9653万元，居全省首位。完成劳务输出107万人次，实现劳务收入52亿元。社会保障体系进一步完善。全市企业养老、医疗保险参保人数分别达到30.5万人和28.5万人，比上年增加3.9万人和0.5万人；参加失业保险36.2万人。提高了最低生活保障标准，五县由100元提高到120元，五区由130元提高到150元。经济适用房竣工21.8万平方米，为2074户城市中低收入家庭解决了住房困难问题。加大农村困难群体救助帮扶力度，提高了农村特困人口最低补助标准和“五保户”集中供养率。社会大局保持稳定。全面构建社会治安防控体系，开展打黑除恶和打击“两抢一盗”活动，打掉涉黑涉恶犯罪团伙64个，有力打击震慑了犯罪。建立健全矛盾纠纷排查调处机制，信访秩序进一步规范。严格落实安全生产责任制，全年没有发生重特大事故，各类伤亡事故、经济损失分别下降25.6%和20.1%。深入开展全民国防教育和“双拥”共建活动，促进了军政、军民团结。认真开展商业贿赂专项治理，反腐倡廉工作取得新进展。

　　各位代表：

　　过去的一年，是我市近年来经济社会发展最快最好的一年，我们实现了“十一五”规划、郑汴一体化发展和新农村建设的扎实起步。这些成绩的取得，得益于省委、省政府和市委的正确领导，得益于市人大、市政协的监督、支持和帮助，凝结着全市人民的心血和汗水。在此，我代表市人民政府，向全市广大工人、农民、知识分子、干部，向驻汴解放军、武警官兵和公安民警以及各界人士致以崇高的敬意！向所有关心、支持开封发展的同志们、朋友们表示衷心的感谢！

　　在肯定成绩的同时，我们也清醒地看到，我市与先进地区的差距仍然较大，前进道路上还有不少困难和问题。一是产业结构不优，一产比重高于全省11.4个百分点，规模以上工业增加值只占生产总值的21.2%，低于全省12.1个百分点。二是经济增长方式粗放，自主创新能力不强，经济运行质量和效益不高，税收占生产总值的比重低于全省平均水平。三是固定资产投资不足，尤其是工业投入不足的问题仍很突出，随着建设用地趋紧等因素的制约，固定资产投资保持较高增幅难度加大。四是“三农”问题仍很突出，农民增收的长效机制还没有真正建立起来。五是财政基础薄弱，政府对社会事业等公共投入不足，社会保障能力不高，影响社会稳定的因素还比较多。六是影响持续发展的体制性机制性障碍尚未完全消除，一些地方和部门仍存在形式主义和官僚主义等现象，经济发展环境有待进一步优化。对这些问题，我们要高度重视并在今后工作中认真加以解决。

　　二、2007年工作的总体要求和任务

　　2007年是贯彻落实市九次党代会精神的第一年，也是全面贯彻落实科学发展观，建设富裕、民主、文明、和谐新开封的关键一年。今年政府工作的总体要求是：以邓小平理论和“三个代表”重要思想为指导，以科学发展观统领经济社会发展全局，认真贯彻落实市九次党代会精神，围绕建设和谐社会和实现开封复兴的总目标，抓住中部崛起和郑汴一体化发展机遇，强力实施工业强市、开放引进等六大战略，着力调整经济结构和转变经济增长方式，着力加强资源节约和环境保护，着力推进改革开放和自主创新，着力促进社会发展和解决民生问题，促进老工业基地向新型工业化城市跨越、传统农业向现代农业跨越、文化旅游大市向文化旅游强市跨越，努力实现经济社会又好又快发展。

　　今年全市经济社会发展的预期目标是：生产总值增长13%以上，其中第一、第二、第三产业增加值分别增长6%、17%和16%；一般预算收入增长16%；全社会固定资产投资增长22%；社会消费品零售总额增长14%；外贸出口增长15%，实际利用外资增长30%；居民消费价格上涨3%以内；城镇居民人均可支配收入增长8%，农民人均纯收入增长5%；单位生产总值能耗降低3%；化学需氧量、二氧化硫排放量分别减少3.5%和1.1%；新增城镇就业6万人，城镇登记失业率控制在4.5%以内；人口自然增长率控制在5.5‰以下。

　　重点抓好以下十个方面的工作：

　　(一)强力推进工业强市战略，构筑经济发展的新格局。充分利用我市现有工业基础，抓住我省建设郑汴洛工业走廊的机遇，认真研究并用足用活国家对老工业基地的各项优惠政策，加大投资力度，优化产业布局，促进产业结构优化升级。突出项目建设，着力建设一批工业区、形成一批产业集群、培育一批骨干企业、打造一批知名品牌，全面提升我市工业经济的竞争力，推进老工业基地向新型工业化城市跨越。加快工业区建设，打造工业发展平台。进一步搞好规划，完善政策，创新体制，优化环境，加强基础设施建设，改善项目落地条件，推动项目向工业区集聚，把工业区建设成为全市的产业基地、创新基地、财源基地和就业基地。强力推进杏花营高新技术工业区、边村重工业区、汪屯精细化工区、黄龙工业区和尉氏—通许—杞县—兰考纺织产业聚集带建设，支持五县工业区加快发展，形成市、县工业区和优势产业带竞相发展的良好局面。发挥开发区体制、政策、区位等优势，加大招商引资力度，努力把开发区建成对外开放的窗口、产业聚集的公共平台、市级工业的重要支撑。培育优势特色产业，促进产业集群发展。加强产业调研，制定纺织、食品、化工、医药、机械设备制造等五大优势产业以及空分、阀门、空间结构、预应力、汽车配件、汴绣等产业集群发展规划。完善产业集群和中小企业发展的各种配套政策，加大各种生产要素的投入力度，积极争取3平方公里的空分特色产业园区列入国家火炬计划，促进我市工业支柱产业的形成和产业集群的发展。建立产业协会，加强行业自律，提高产业整体竞争力。继续扩大招商引资，实行项目带动。根据我市的资源优势和产业发展方向，认真做好项目的筛选、包装和推介，着力引进国内外知名企业和知名品牌，借助其优质资本和人才、市场、管理优势，带动我市工业又好又快发展。要切实搞好招商引资项目的跟踪服务，在谈项目抓签约，签约项目抓落地，落地项目抓进度，建成项目抓投产。今年我市确定实施70个重点工业项目，总投资84.4亿元。继续实行市领导联系重点企业和重点项目制度，对中原皓月综合加工、京源公司2×600MW发电机组、晋开公司“1830”工程、平煤集团开封东大化工公司年产6万吨有机硅和15万吨离子膜烧碱改扩建、永煤集团铁塔公司橡胶工业园等重大项目，由市领导牵头，成立专门工作组，及时解决项目建设中的问题。要充分挖掘存量土地潜力，加大土地储备、开发复垦力度，确保重点项目建设用地需要。加强企业诚信建设，积极推进银企合作，拓宽项目融资渠道，引导金融资本、市外资本和民间资本投向重点项目。坚持扶优培强，培育一批骨干企业。围绕优势产业和特色产品，培育壮大现有企业，对发展潜力大、科技含量高的高成长性企业，要制定政策，及时解决发展中的实际问题，支持其尽快做大做强，力争在3—5年内培育出一批产值超10亿元、超20亿元、超30亿元的大企业，努力实现1—2家企业上市融资。积极实施名牌战略，鼓励企业联大联强、联名联优，争创名牌企业，发展名优产品，发挥名牌效益。

　　(二)大力发展县域经济，迈出新农村建设的新步伐。全面落实促进县域经济发展的各项政策措施，激发县域经济发展活力，促进县域经济晋位升级，扎实推进社会主义新农村建设。坚持用工业化的理念发展农业，促进传统农业向现代农业跨越。加快工业经济发展，壮大县域经济实力。始终坚持把发展工业作为壮大县域经济的根本途径，以工业化带动城镇化和农业现代化。各县要发挥优势，突出特色，着力培育1—2个特色产业。大力发展农副产品加工和深加工，积极培育龙头企业，推广公司＋基地＋农户的经营模式，拉长产业链条，推进农业产业化进程。大力发展现代农业，提高农业综合生产能力。加强农田水利基本建设，提高农业技术装备水平，加强农业科研和技术推广，加快优质专用小麦等大宗优质农产品生产和加工基地建设，积极发展优质花生、汴梁西瓜、杞县大蒜等特色农业，推进水产养殖和菊花生产规模化、产业化。推广健康养殖方式，发展规模养殖和畜禽养殖小区，狠抓畜产品加工、流通和质量安全，做大做强畜牧业。积极发展以速生丰产林为主的工业原料林，加快林业产业化步伐。发展和完善各类专业合作经济组织，壮大农民经纪人队伍，提高农民进入市场的组织化程度。加强农产品市场体系建设，建立大宗农产品应急销售机制和网络，切实解决西瓜、辣椒等大宗农产品卖难问题。加大对“三农”的投入，改善农村生产生活条件。抓住国家和省加大对“三农”投入的有利时机，积极争取上级资金，增加市、县财政投入，引导金融机构加大对“三农”的信贷投放，加快农村饮水、道路、供电、邮政、通信、沼气等基础设施建设。强化农民职业技能培训，提高劳务输出的层次和水平，拓宽农民增收渠道。实施“43300”工程，扎实推进新农村建设试点工作。按照生产发展、生活宽裕、乡风文明、村容整洁、管理民主的总体要求，加强扶持，分类指导，积极探索符合各县区实际的新农村建设模式。在原有31个示范村和300个整治村的基础上，再增加12个示范村。示范村重点培植发展新产业，完成“三清四改”(清垃圾、清污泥、清路障，改水、改厕、改圈、改路)，实现“六通一气”(通水、通路、通电、通广播电视、通电话、通宽带，沼气入户率达到80%)；整治村要巩固“三清”和文明村镇创建成果，沼气入户率达到20%。

　　(三)全面提升文化旅游产业和现代服务业水平，培育经济发展的新优势。以文化旅游资源为依托，以培育文化旅游龙头企业为重点，以发展现代服务业为支撑，努力打造中部地区旅游休闲之都，加快由观光游向休闲度假游转变，推进文化旅游资源大市向文化旅游强市跨越，全面提升第三产业发展水平。整合开发文化资源，大力发展文化产业。振兴传统文化产业，发展新兴文化产业，着力实施文化精品项目，发展龙头骨干企业，培育知名文化品牌，建设文化产业基地，把文化产业打造成为我市经济社会发展的新亮点和国民经济的支柱产业之一。发挥我市景观文化、府衙文化、饮食文化、民俗文化、宗教文化等品牌优势，促进文化产业与旅游产业的互动发展。加快建设汴绣、木版年画、官瓷等生产园区，推进我市特色文化产品的产业化、基地化生产。发展网络服务、广告会展、文化创意等新兴文化产业，支持新闻出版、广播影视、文艺演出等主导文化产业，促进重点文化企业做大做强。推动文化产业与高新技术相结合，完成市区有线电视由模拟向数字整体转换。加强文化产业项目库建设，搞好文化项目推介和招商工作，培育外向型文化企业。积极推进文化体制改革，全面完成经营性文化事业单位转企改制，鼓励和引导非公有制资本进入文化领域，促进民营文化企业健康发展。策划举办第一届中国收藏文化(开封)论坛活动，提高开封文化知名度。实施旅游带动战略，加快旅游产业化步伐。以建设具有较强吸引力的国际文化旅游城市为目标，以宋都古城复建工程为主线，理顺体制，创新机制，整合资源，市场运作，着力打造宋都、菊乡和水城三大品牌，培育旅游龙头企业，推进旅游产业化进程，把旅游业发展成为我市国民经济的支柱产业之一。以资产为依托，以利益为纽带，以所有权、管理权和经营权分离为突破口，积极推进旅游景区(点)改组、改制。强化旅游推介，大力开展旅游招商，引进国内外知名企业，参与我市旅游业开发。培育壮大具有较强带动能力的旅游龙头企业和企业集团。围绕吃、住、行、游、购、娱，规划建设若干特点突出、吸引力强的休闲旅游集聚区，促进开封旅游由观光游向休闲度假游转变。加快金明池等重点旅游项目建设，完成水系二期工程，做好沿岸和水上旅游项目开发，建成贯穿景区之间的水上旅游观光带。启动实施开封文化夜市建设项目，推出一批特色表演节目，加强夜间景观建设，打造夜游开封品牌。大力发展现代服务业，全面提升第三产业发展水平。坚持市场化促进、信息化支撑和法制化保障，分层次、有重点地推进现代服务业领先增长，努力构建高增值、强辐射、广就业的现代服务业体系，实现三次产业融合与互动发展。优化服务业网点布局，鼓励支持传统服务业不断扩大规模、提升档次。发展连锁经营、物流配送、电子商务等新型流通业态，培育大型商业集团。整合政府、社会和社区资源，大力发展家政服务、物业管理、养老托幼、医疗保健等社区服务，构建覆盖全市的多元化社区服务体系。实施“万村千乡市场工程”，发展连锁农家店、农资配送中心、农资超市等，探索发展农村商贸服务业的新机制。积极发展信息、中介、房地产、银行、证券、保险、策划、法律等服务业，推动商务服务方式创新。

　　(四)破除体制机制障碍，实现改革开放的新突破。进一步加大改革力度，提高对外开放水平，增强经济社会发展活力。深化企业改革。坚持以盘活存量、扩大增量为目标，以妥善安置职工为前提，以引进外来资本为主导，强力推进以产权制度改革为主要内容的国有企业改革，积极推进非工业企业改革。改制不规范、不彻底、不成功的企业，要寻求新的形式继续深化改革，尽快建立现代企业制度。深化农村综合改革。以转变职能为重点，深化乡镇机构改革。以落实教育经费保障机制为重点，推进农村义务教育改革，确保各级财政承担的公用经费按规定增加、编制内教师工资按现行标准及时足额发放。推进县乡财政管理体制改革，完善乡财县管制度，增强财政保障能力。深化人事和收入分配制度改革。认真组织实施公务员工资制度改革和规范津贴补贴工作。全面推行事业单位人员聘用、岗位管理和收入分配制度改革，继续推进经营服务型事业单位转企改制和科研事业单位管理体制改革。加快交通、医疗卫生等改革，力争取得阶段性成效。继续实施开放引进主战略。改进和创新招商方式，更加注重引进项目的产业方向、技术含量、环保要求、投资规模和经济效益，提高招商引资的质量和水平。加强项目库、商情库和信息发布平台建设，谋划、推介和实施一批关系全局和长远发展的重大项目。积极参加中部投洽博览会、厦交会等活动，办好中国开封第25届菊花花会，组织好赴杭州、台湾等地的招商活动，全年引进市外资金100亿元以上。加大引进外资的力度，确保今年实际利用外资4000万美元以上。发展外向型经济，扩大机电、医药、新材料等高新技术产品出口，重点扶持兴化精细化工厂、特耐集团、三环桐材等一批出口骨干企业。加强对外劳务合作，扩大海外工程承包和劳务输出规模。优化经济发展环境。着眼于建立优化经济发展环境的长效机制，进一步创新政务服务机制。继续推行项目代理制，为外来投资者提供优质便捷服务。逐步实行涉企“一费制”，减轻企业负担。整顿和规范市场经济秩序，加强诚信体系建设，打造诚信开封。深入开展万人评议机关活动，着力解决服务意识不强、工作效率不高的问题，严肃查处影响和破坏投资环境的行为。

　　(五)加快郑汴一体化发展步伐，打造加速发展的新平台。抓住郑汴一体化发展的重大机遇，根据区域对接、资源共享、优势互补、错位发展的总体思路，强力开发汴西新区。按照集高新产业、居住、办公、商贸、教育、休闲为一体的功能定位，加快新区基础设施建设，全面展开产业布局，加强生态环境整治，把汴西新区建设成为功能高度集中、基础设施配套完备、产业发展健康有序、用地集约、环境友好、可容纳80万人口的现代化、生态型的新城区。建立高效协调机制和管理体制。加强与省直有关部门及郑州市的协调合作，理顺汴西新区建设管理体制，形成集中、统一、高效的管理运行机制，确保汴西新区建设快速运转。加快基础设施建设。进一步完善政策，建立多元投资机制，加快新区基础设施建设，提高新区综合承载能力。继续抓好已开工各项基础设施建设；建成郑开大道两侧防洪除涝开封段工程；投入4亿元，建设魏都路西延、宋城路西延、区内1号路、4号路、5号路、8号路等道路，同步推进供水、排水、供电、通讯、热力、燃气、污水处理等市政设施建设；完成2×300MW热电联供和马家河北支、运粮河景观整治等项目的前期工作，加快杏花营水厂、污水处理厂的项目论证，为实现汴西新区“三年树形象、五年成规模”奠定坚实基础。全力推进郑开大道两侧项目建设和产业布局。抓紧编制郑开大道两侧用地控制性详细规划及汴西新区专项规划，尽快在郑开大道两侧展开工业、商住、金融、教育、生态等建设，带动汴西新区的建设与发展。重点抓好项目的落地率、开工率、完工率和投产率，力争起步区面积达到5平方公里。汴西新区产业发展要按照规划确定的产业分区，认真筛选项目，积极引导高新技术项目、清洁环保项目、投资强度大的项目向汴西新区集中，逐步形成郑汴紧密相连的产业带，切实把汴西新区建设成为中原城市群核心区的先导区、高新技术产业的集聚区、全省城乡协调发展和集约用地的示范区。

　　(六)加强城市规划、建设与管理，促进城镇化水平的新提高。按照“古今文明交相辉映、新老城区各展风采、宋都古城特色浓郁”的城市发展总体布局，坚持“保护、修缮、适度开发老城区，高标准建设新城区”的方针，完成城市总体规划修编工作，加快城市建设和改造步伐，推进城镇化进程，全市城镇化率提高1.5个百分点。实施宋都古城风貌保护与重现工程。抓好水系二期、延庆观顶升扩建、鼓楼复建等重点工程建设。整治和规范商业街区，规划建设停车场、垃圾中转站、公共厕所等公共设施，加快背街改造步伐，争取开工建设金耀路，逐步改善城市交通状况和人居环境。支持老城区机关、企业和人口外迁，降低人口和建筑密度，促进老城区的保护和修缮。同时，进一步完善东区基础设施，搞好东区开发和建设，提高城市发展规模和档次。加快县城和小城镇建设。加强城镇的规划、建设、管理和经营，多元投资，加快县城和小城镇的建设步伐，突出抓好8个省重点镇的建设和发展。坚持把县城和小城镇建设与市场建设、产业集群发展结合起来，增强城镇综合经济实力和辐射力，引导人口向城镇集中、产业向园区集中，以人口和产业聚集带动县城规模扩大和中心城镇发展。深化户籍管理制度改革，为农民进城创业、就业、安居营造良好的社会环境。加大城市管理力度。进一步整合和理顺城市管理体制，创新管理模式，充分发挥各区、街道和社区在城市管理中的作用。加强社区建设，完善社区服务功能，健全社区工作机制，提高社区自我管理水平。继续推进城乡结合部环境综合整治，加快“城中村”改造步伐。巩固“创三城”成果，争创国家园林城市、卫生城市和省级文明城市。

　　(七)大力发展非公有制经济，形成全民创业的新局面。非公有制经济是工业强市的主力军，全民创业是经济发展的强大动力。要毫不动摇地支持非公有制经济加快发展，大力推进全民创业。放手发展非公有制经济。进一步加大对非公有制经济的政策扶持力度，支持非公有制经济参与国有经济战略性调整和国企重组改制，进入金融服务、公用事业、基础设施以及法律法规未限制的一切领域，支持有条件的非公有制企业做大做强。建立民间风险投资和融资中介机构，为非公有制企业搞好融资担保、创业辅导、教育培训等方面的服务和支持，营造有利于非公有制企业发展的良好环境。大力推进全民创业。营造全民创业的浓厚氛围，弘扬创业精神，强化创业理念，培育创业文化，激发全社会的创造活力，以创业推动发展，以创业带动就业，以创业促进和谐。切实改进创业服务，加强政策和信息引导，制定和完善全民创业政策措施，在降低门槛、土地使用、信贷税收等方面创造平等竞争环境，推动创业成功。

　　(八)加强资源节约和环境保护，在建设资源节约型、环境友好型社会上取得新进展。把节约能源资源和保护生态环境作为加快经济结构调整、促进经济增长方式转变的重要抓手，采取有效措施，确保完成节能降耗和污染减排任务。强力推进节能降耗。建立健全节能降耗目标责任制，完善能耗指标公报和重点用能单位能源利用状况报告制度，着力抓好高耗能行业和重点耗能企业的节能降耗。加快推进节能技术进步，加快先进节能技术、节能产品的研发和推广应用。大力发展循环经济，构建节约型产业结构，促进资源循环利用。强化土地节约集约利用。加强耕地特别是基本农田保护，严格耕地占补平衡，继续推进“空心村”、砖瓦窑场、废弃地三项整治。实行经营性土地招标拍卖挂牌出让制度，严格核定项目用地标准，减少企业非生产性用地。优化工业用地布局，以各类开发区、工业区为重点，推广使用多层标准厂房，提高投资强度。加强环境保护和生态建设。全面落实环境影响评价制度、污染物排放总量控制制度和环境目标责任制。做好城市大气、水、噪声、垃圾污染和农村污染源的防治工作，继续严查“十五小”、“新五小”和其他违反国家产业政策的污染企业。以创建绿色社区、生态示范乡镇活动为载体，建设生态城市，打造绿色开封。

　　(九)建设创新型开封，为经济社会发展注入新动力。以提高核心竞争力为目标，把提高自主创新能力作为推动“科教兴汴、人才强市”的战略基点，作为调整产业结构、促进增长方式转变的中心环节，推动经济增长由资源依赖型向创新驱动型转变。着力提高自主创新和吸收再创新能力。优化科技资源配置，加大科研投入，加强知识产权保护，加速科技成果的推广和应用。大力发展高新技术产业，积极引进国内外先进技术，加大企业技术改造力度，培育一批具有自主知识产权的优势产品和高新技术产业，力争在纺织、食品、精细化工、机械设备制造等领域突破若干关键技术，掌握一批核心技术，打造若干在国内国际市场上具有较强竞争力的企业和企业集团。组织实施技术创新引导工程，鼓励支持高新企业、大中型企业和有条件的民营科技企业建立工程技术中心和重点实验室。加强与中国农大、河南大学等省内外高校和科研院所的共建合作，促进产学研联盟。加快创新型人才队伍建设。树立人才是第一资源的观念，进一步完善政策，培养和引进一批具有较强创新能力的学术技术带头人，一批懂科技、会经营、善管理的企业家，一批专业化、高技能的实用人才。完善人才选用、职称评聘等制度，营造有利于各类人才脱颖而出的良好环境。

　　(十)更加注重解决民生问题，建设文明和谐的新开封。坚持以人为本，以解决人民群众最关心、最现实的问题为重点，加快推进和谐开封建设，提高人民幸福指数。全面发展各项社会事业。加大对教育的投入力度，优化整合教育资源，改善办学条件，提高教育质量。巩固提高义务教育，切实加强素质教育。大力发展职业教育，加快职业教育中心建设。积极发展高等教育，支持河南大学、黄河水利职业技术学院、开封大学、开封教育学院等院校发展。加强市场监管，保证食品药品安全。发展体育事业，广泛开展全民健身活动，办好市七届运动会。认真落实计划生育基本国策，继续稳定低生育水平，提高出生人口素质，统筹解决出生人口性别比偏高、流动人口管理、人口老龄化等问题。积极发展慈善事业和残疾人事业。加强社会主义精神文明建设。以开展社会主义荣辱观教育为核心，加强世界观、人生观、价值观教育，深入开展知荣辱、讲正气、树新风、促和谐活动。认真贯彻公民道德建设实施纲要，切实抓好未成年人思想道德建设和大学生思想政治教育，推进社会公德、职业道德和家庭美德建设。深入开展窗口行业规范管理优质服务竞赛活动，继续开展文化、科技、卫生“三下乡”和科教、文体、法律、卫生“四进社区”等活动，提升公民素质和社会文明程度。大力推进平安开封建设。完善社会治安防控体系，加强农村和社区警务工作，深入开展严打专项斗争，预防和减少各类刑事犯罪。建立健全维护稳定的工作机制，依法规范信访秩序。全面落实安全生产责任制，坚决遏制重特大事故的发生。建立健全社会预报预警和应急救援体系，提高对自然灾害和突发性事件的处置能力。认真贯彻落实党的民族宗教政策，广泛开展民族团结进步创先活动，促进各民族群众和谐共处、共同发展。开展国防教育，支持驻汴解放军和武警部队建设，抓好人民防空和民兵预备役工作。深入开展双拥共建活动，巩固和发展军政军民团结。充分发挥工会、共青团、妇联等人民团体的桥梁纽带作用。加强外事、侨务工作，继续做好统计、审计、电力、电信、邮政、烟草、物价、气象、地震、对台、档案、地方志、社会科学等工作。

　　着力办好关系全市人民切身利益的15件实事。1.加大对农民的补贴力度。继续实施对种粮农民直接补贴、农业生产资料综合直补等7项补贴，确保补贴金额高于上年。2.继续改善农村基本生产生活条件。加强农村公路建设、管理与养护，解决16万人的农村安全饮水问题，新发展沼气用户2万户。3.巩固提高农村义务教育水平。全面免除农村义务教育阶段学杂费，继续对困难家庭学生免费提供课本并补助寄宿生生活费。用两年时间把贫困地区农村中小学课桌更新配置一遍。全面完成农村中小学现代远程教育工程。4.提高城区中小学公用经费保障水平。从2007年起，城区中学、小学公用经费分别由20元/生年、12元/生年提高到100元/生年和60元/生年。5.缓解群众看病难、吃药贵问题。加强公共卫生体系和农村卫生基础设施建设，开工建设市一中医院病房楼。扩大新型农村合作医疗覆盖面，新增通许、杞县、金明区三个新型农村合作医疗试点县(区)。加快建立特困职工、弱势群体大病救助机制。继续降低药品价格，整顿医疗服务价格。6.繁荣农村文化事业。加大对公益文化事业的扶持力度，加快各县群艺馆、文化馆、图书馆和乡村文化站、文化大院建设，实施农村电影放映工程，丰富农民文化生活。7.做好农村劳动力转移就业工作。继续实施农村劳动力技能就业计划、农村劳动力培训阳光工程，完成农村劳动力培训15万人，其中免费培训5万人，实现劳务输出110万人次以上、务工收入60亿元以上。8.及时解决“零就业”家庭问题。完善和落实就业再就业优惠政策，多渠道开发就业岗位，全年城镇新增就业6万人，实现下岗失业人员再就业3万人，其中“4050”人员1万人；确保每个有就业能力人员的家庭至少有1人就业。9.扩大社会保障范围。扩大社会保险覆盖面，尽快使养老、医疗、失业、工伤、生育等保险覆盖到各类企业。建立完善机关事业单位养老保险制度，适时提高城镇最低工资标准。10.加大对城乡困难群体的救助力度。完善农村低保制度，提高最低保障标准，农村低保由每人每月20元提高到30元。农村五保户集中供养率提高到30%。增加对城乡医疗救助的资金补助，确保补助金额高于上年。加大扶贫开发力度，完成3万人脱贫和解决温饱。11.加快经济适用房和廉租房建设。改进和规范经济适用住房制度，新开工建设经济适用房30万平方米，解决3000户以上城市低收入家庭住房问题。从今年2月起，实行廉租住房制度，使城镇最低收入家庭享受廉租住房或住房补贴。12.加快老城区背街改造步伐。改造徐府街、天地台南北街等城市背街26条，解决部分街道路面不平、道路积水、路灯不亮问题。13.增设城市环卫设施。新建生活垃圾中转站4座、水冲式公厕10座，解决部分社区、广场、夜市垃圾中转和群众如厕问题。14.提高城市公交运营能力。更新13路、24路公交车32台，新开30路(豪德贸易广场—公交三公司)、31路(金明广场—火车站)、32路(河大新校区—火车站)公交线路，改善居民出行条件。15.进一步加强污染防治。将惠济河、贾鲁河作为流域水污染综合整治的重点，使河流水质达到省政府确定的2007年出境断面水质目标要求。加强饮用水源保护，确保饮用水源安全。建成投用东区、五县污水处理厂和市垃圾处理厂、医疗废物处理厂，改善人民群众生产生活环境。

　　各位代表：

　　人民群众是我们的“衣食父母”，为人民服务是党和政府的根本宗旨。我们一定要牢固树立执政为民的理念，怀着深厚的感情为群众做好事、办实事、解难事。尽管我们不能在短时期内把所有问题都解决好，但我们必须把工作精力尽可能地向这方面集中，把财力、物力最大限度地向这方面倾斜，从解决群众最急需解决的问题入手，从能够办好的事情做起，抓住不放，持之以恒，争取每年都办成几件与群众利益密切相关的好事、实事，让广大人民群众得到更多实惠，共享改革发展成果！

　　三、加强政府自身改革和建设

　　建设为民、务实、清廉、高效的政府，打造有利于开封发展的良好环境，是人民群众的迫切要求，也是全面完成今年各项目标任务的重要保证。要以转变政府职能为核心，以提高政府效能、增强政府执行力和公信力为出发点，大力推进体制创新、机制创新、制度创新和管理创新。要自觉接受人民代表大会和人民政协的监督，广泛听取各民主党派、工商联和无党派人士的意见和建议，办好各项提案议案，推进决策的科学化、民主化、法制化。要按照建设服务型政府的要求，努力做到以下几点：

　　一是为民。坚持以民为本，把维护好、实现好、发展好人民群众的根本利益作为各项工作的立足点、出发点和根本点，多做打基础、管长远、惠百姓的工作。切实搞好经济协调和市场监管，更加注重社会管理和公共服务。认真解决土地征用、房屋拆迁和企业改制中损害群众利益的突出问题，特别对群众就业、就医、住房、子女上学等实际困难和问题，要千方百计加以解决，真正做到察民情、解民忧，言必信、行必果，切实取信于民。

　　二是务实。牢固树立科学发展观和正确的政绩观，把发展的热情、科学的态度和务实的作风统一起来，继续弘扬焦裕禄精神，大兴求真务实之风，力戒心浮气躁，防止急功近利，克服官僚主义和形式主义。坚持讲实话、鼓实劲、干实事、求实效，各项工作都要经得起实践的检验、群众的检验和历史的检验。大力精简会议、文件，把更多的时间和精力用在调查研究和工作落实上。强化工作责任，加强督促检查，实行全程跟踪、即时监控，确保各项具体工作措施的落实。

　　三是清廉。牢记“两个务必”，大力发扬艰苦奋斗和勤俭节约精神，坚决抵制拜金主义、享乐主义和奢靡之风。严格财政支出管理，进一步规范公务接待行为，建设节约型机关。全面落实党风廉政建设责任制，教育政府工作人员严于律己，克己奉公，增强为民服务意识，提高拒腐防变能力。加大标本兼治、综合治理和查处违法违纪案件的力度，加强行政效能监察、审计监督和行风建设，把廉政建设和反腐败工作推向深入。

　　四是高效。完成今年各项工作任务，要求我们必须进一步提高工作效率，加快工作节奏。要切实加强学习，不断提高驾驭工作的能力和水平。要始终保持奋发有为、昂扬向上的精神状态，发扬只争朝夕、人一我十的精神，对关系全市发展全局的重大问题和人民群众切身利益的实际问题，主动研究，主动协调，急事急办，特事特办。要继续深入开展效能革命，创新行政方式和运行机制，规范工作秩序，提高政府工作效率，为开封发展创造良好的政务环境、法制环境、社会环境和人文环境。

　　各位代表！

　　新的一年，新的征程。让我们紧密团结在以胡锦涛同志为总书记的党中央周围，高举邓小平理论和“三个代表”重要思想伟大旗帜，全面贯彻落实科学发展观，开拓创新，扎实工作，努力开创我市经济社会发展新局面，为加快开封复兴，建设富裕、民主、文明、和谐的新开封而努力奋斗！
